# Acute Kidney Injury in Hospitalized Patients with Community-Acquired Pneumonia: Frequency, Factors Associated with AKI, and Clinical Outcomes

**DOI:** 10.3390/jcm15197616

**Published:** 2026-10-01

**Authors:** Elvan Senturk Topaloglu, Aziz Gumus, Eray Halisdemir, Omer Topaloglu, Hasan Veysel Keskin, Neslihan Ozcelik, Songul Ozyurt, Unal Sahin

**Affiliations:** 1 Department of Pulmonology, Faculty of Medicine, Recep Tayyip Erdogan University, 53100 Rize, Turkey; elvan.senturktopaloglu@erdogan.edu.tr (E.S.T.); aziz.gumus@erdogan.edu.tr (A.G.); erayhalisdemir@live.com (E.H.); neslihan.ozcelik@erdogan.edu.tr (N.O.); songul.ozyurt@erdogan.edu.tr (S.O.); unal.sahin@erdogan.edu.tr (U.S.); 2 Department of Thoracic Surgery, Faculty of Medicine, Recep Tayyip Erdogan University, 53100 Rize, Turkey; 3 Department of Pulmonology, Ahi Evren Thoracic and Cardiovascular Surgery Training and Research Hospital, Trabzon University, 61040 Trabzon, Turkey; keskinh.veysel@gmail.com

**Keywords:** community-acquired pneumonia, acute kidney injury, blood urea nitrogen, chronic kidney disease, confusion, intensive care unit, hospitalization

## Abstract

**Background:** Community-acquired pneumonia (CAP) is a common infectious disease associated with substantial morbidity and mortality. Acute kidney injury (AKI) is a frequent complication of CAP and may adversely affect clinical outcomes. This study aimed to determine the frequency of AKI and identify factors associated with AKI in hospitalized patients with CAP. **Methods:** This retrospective cohort study was conducted at a tertiary care university hospital in Türkiye and included patients aged >18 years hospitalized with CAP between July 2024 and July 2025. Only patients initially admitted to the general ward were included. Patients receiving dialysis for end-stage renal disease, those with active malignancy, those receiving immunosuppressive therapy, and those with incomplete data were excluded. Demographic characteristics, vital signs, laboratory findings, comorbidities, smoking status, and medication histories were recorded. AKI was defined according to KDIGO criteria using the closest clinically stable pre-admission serum creatinine value obtained within 7–365 days before hospitalization as baseline. **Results:** Among 161 patients, AKI was identified in 60 (37.3%), including 43 patients who fulfilled the KDIGO criteria at hospital admission and 17 who developed AKI during hospitalization. Eight patients with AKI (13.3%) required hemodialysis, although none became permanently dialysis-dependent. Patients with AKI had longer hospital stays (*p* = 0.038) and a greater need for intensive care (*p* = 0.028). They were older and had higher respiratory rates, blood urea nitrogen (BUN), and red cell distribution width (RDW) values and lower diastolic blood pressure. DM and CKD were more common in the AKI group. In multivariable analysis, confusion (adjusted OR 5.03, 95% CI 1.01–25.02; *p* = 0.048), CKD (adjusted OR 6.00, 95% CI 1.28–28.13; *p* = 0.023), and higher BUN levels (adjusted OR 1.095 per 1 mg/dL increase, 95% CI 1.046–1.146; *p* < 0.001) were independently associated with AKI. These associations were retained in a Firth penalized logistic regression sensitivity analysis. **Conclusions:** AKI was common among hospitalized patients with CAP and was associated with longer hospital stay and greater intensive care utilization. Confusion, pre-existing CKD, and elevated BUN levels were independently associated with AKI, whereas DM was not independently associated after multivariable adjustment. Given that most AKI cases were already present at hospital admission, these findings should be interpreted as associations rather than predictors of incident AKI.

## 1. Introduction

Community-acquired pneumonia (CAP) is one of the most frequently encountered infectious diseases worldwide and continues to represent a major public health problem in terms of both morbidity and mortality [1,2]. Particularly in older patients and in the presence of accompanying comorbid diseases, the resulting systemic inflammatory response may lead to multiple organ dysfunction and substantially worsen the clinical course of the disease. Among these organ dysfunctions, acute kidney injury (AKI) is especially prominent. AKI is a common extrapulmonary complication of CAP; in a large prospective multicenter cohort of hospitalized patients with CAP, AKI occurred in approximately one-third of patients [3]. The clinical spectrum of CAP ranges from disease managed on general wards to severe illness requiring intensive care and ventilatory support [4,5,6]. In patients with CAP, the presence of AKI has additionally been associated with adverse clinical outcomes, including reduced survival [3].

AKI is a clinical syndrome characterized by a sudden decline in glomerular filtration rate, an increase in serum creatinine levels, and/or decreased urine output. In patients with CAP, AKI may already be present at hospital admission and has been associated with adverse prognosis [7]. It is currently classified according to the Kidney Disease: Improving Global Outcomes (KDIGO) criteria, which represent the most widely accepted definition system [8]. Numerous mechanisms are involved in its pathophysiology, including renal hypoperfusion, endothelial dysfunction, inflammatory cytokine activation, oxidative stress, and tubulointerstitial injury. The pathophysiology of infection-associated AKI is multifactorial, and these mechanisms may coexist rather than occur in isolation. Accurate recognition and characterization of AKI in patients with acute infection are important because its presence is associated with adverse clinical outcomes.

AKI is frequently encountered in hospitalized patients with acute infection, making the characterization of its frequency and associated clinical factors particularly relevant in inpatient populations. Previous studies of CAP-associated AKI have included heterogeneous populations, including large mixed hospitalized cohorts and patients with AKI already present at admission [3,7]; however, data specifically describing the frequency of AKI and its associated clinical factors among patients initially admitted to general wards remain limited. Accordingly, the present study focuses on hospitalized patients with CAP admitted to general wards, representing a clinically relevant inpatient population encountered in routine practice.

This study aimed to determine the frequency of AKI in patients hospitalized with a diagnosis of CAP and to identify the clinical and laboratory factors associated with the presence of AKI. By focusing on this population, we sought to characterize the clinical profile of CAP patients with AKI and to examine factors associated with AKI without implying prediction of subsequent AKI development.

## 2. Materials and Methods

### 2.1. Study Population and Design

**Figure 1:**
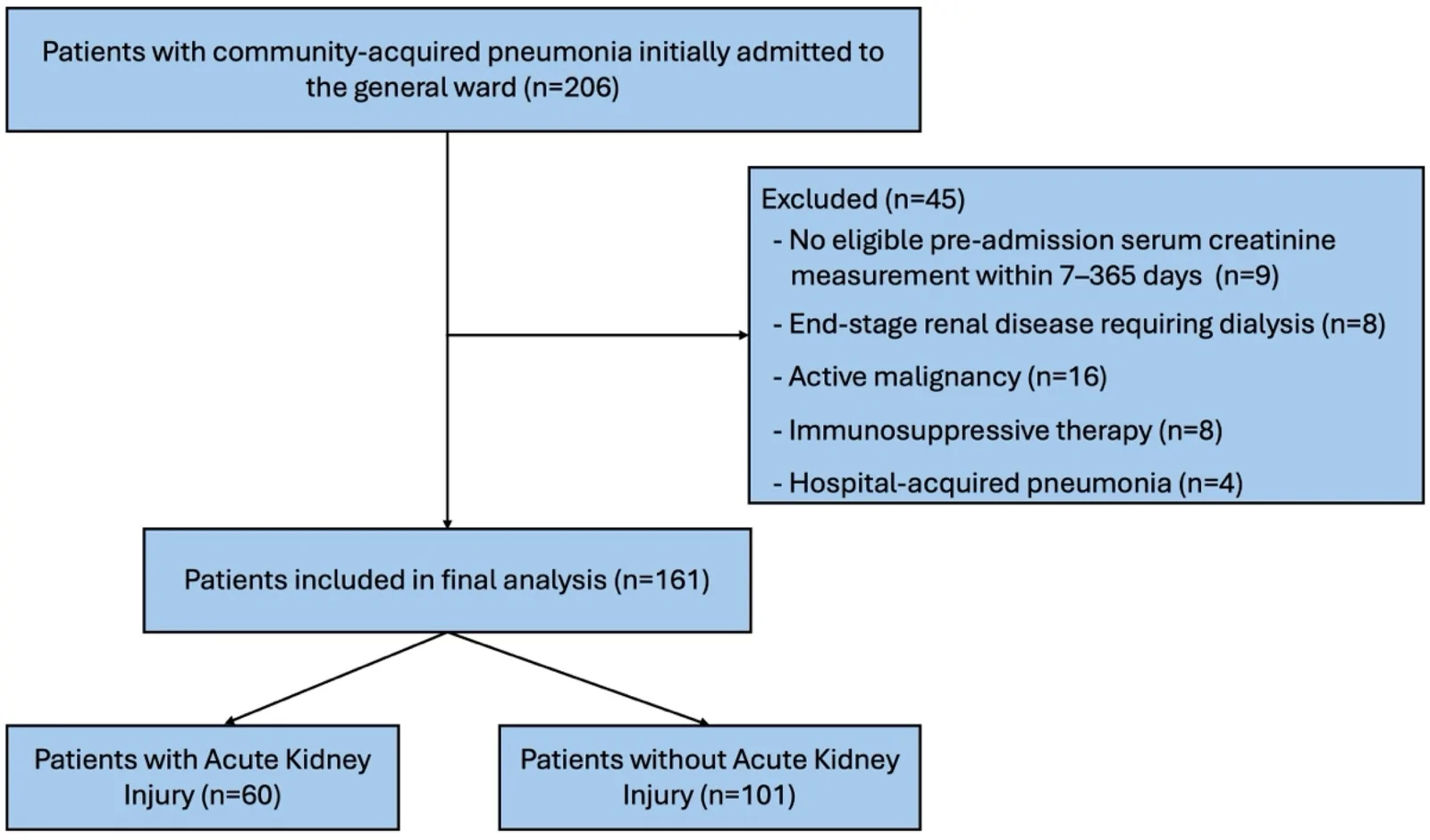
Flowchart of patient inclusion, exclusion, and study grouping.

The initial screening cohort comprised 206 adult patients with CAP who were initially admitted to the general ward between July 2024 and July 2025. Patients admitted directly to the ICU were not included in this screening cohort. Patient data were retrospectively collected from electronic medical records and hospital database systems. Patients receiving regular dialysis due to end-stage renal disease, those with active malignancy, those receiving immunosuppressive therapy, and those diagnosed with hospital-acquired pneumonia were excluded. Patients without an eligible pre-admission serum creatinine measurement within 7–365 days before hospital admission (*n* = 9) were also excluded because baseline renal function could not be reliably determined. All 161 patients included in the final analysis had at least one pre-admission serum creatinine measurement available between 7 and 365 days before admission through the institutional electronic medical record system, including outpatient visits, emergency department admissions, and previous hospitalizations. For patients with more than one measurement within this period, the closest clinically stable value preceding the index hospitalization was used as the baseline serum creatinine, as described below. A flowchart illustrating patient selection, exclusion criteria, and final study grouping is presented in Figure 1. The study protocol was approved by the Ethics Committee of Recep Tayyip Erdoğan University (Approval No. 2025/439; 4 November 2025), and the study was conducted in accordance with the principles of the Declaration of Helsinki.

### 2.2. Definition of CAP

CAP was defined by the presence of a newly developed infiltrate on chest radiography or thoracic computed tomography, together with at least one of the following clinical/laboratory criteria:

1. Recent onset of sputum production, cough, or dyspnea;
2. Peripheral white blood cell counts of >10 × 10^3^/mm^3^ or <4 × 10^3^/mm^3^;
3. Body temperature of >38.0 °C [6].

### 2.3. Definition of AKI

AKI was defined according to the serum creatinine-based KDIGO criteria [8]. AKI was considered present if either of the following criteria was met: (1) an increase in serum creatinine of ≥0.3 mg/dL between the admission serum creatinine and the serum creatinine measured within the subsequent 48 h, or (2) an increase in serum creatinine to ≥1.5 times the pre-admission baseline value within the first 7 days of hospitalization. Baseline serum creatinine was defined as the closest clinically stable serum creatinine measurement obtained within 7–365 days before hospital admission. When multiple eligible measurements were available, the measurement closest to the index admission was selected after review of the corresponding clinical record to confirm that it represented stable renal function. Admission, 48-h, and day-7 serum creatinine values were evaluated for all patients. Peak serum creatinine was defined as the highest value among the admission, 48-h, and day-7 measurements. Patients whose admission serum creatinine already fulfilled the ≥1.5-fold baseline criterion were classified as having AKI at admission; patients who did not meet AKI criteria at admission but fulfilled the KDIGO criteria during the subsequent hospitalization were classified as having incident AKI. Because urine output data could not be reliably obtained owing to the retrospective study design, AKI was diagnosed using serum creatinine-based KDIGO criteria. AKI severity was staged according to KDIGO criteria, and initiation of renal replacement therapy was classified as stage 3 AKI.

### 2.4. Data Collection

The initial clinical evaluation and laboratory data of the patients were obtained based on the records documented within the first 6 h after hospital admission. Demographic characteristics, vital signs, arterial blood gas parameters, biochemical analyses, smoking status, comorbidities, and medication history were recorded. Comorbidities included hypertension, diabetes mellitus, coronary artery disease, congestive heart failure, chronic obstructive pulmonary disease, dementia, cerebrovascular disease, atrial fibrillation, and chronic kidney disease. Pre-admission medication data included angiotensin-converting enzyme inhibitor/angiotensin II receptor blocker (ACE/ARB) and diuretic use. Exposure to contrast media and antibiotic exposure during hospitalization was also recorded and categorized as penicillins, cephalosporins, quinolones, macrolides, and vancomycin. In addition, the CURB-65 score was calculated at hospital admission using its standard components: confusion, BUN > 19.6 mg/dL (equivalent to urea > 7 mmol/L), respiratory rate ≥ 30 breaths/min, systolic blood pressure < 90 mmHg or diastolic blood pressure ≤ 60 mmHg, and age ≥ 65 years [9].

Hypotension was defined as a systolic blood pressure < 90 mmHg and/or a diastolic blood pressure ≤ 60 mmHg at hospital admission. Acidosis was defined as an arterial blood pH < 7.35 at hospital admission.

Diabetes mellitus (DM) was defined as a previously documented diagnosis in the electronic medical records and/or the documented use of glucose-lowering medication before the index hospitalization, consistent with the American Diabetes Association (ADA) Standards of Care [10]. Pre-existing chronic kidney disease (CKD) was identified from a documented diagnosis in the electronic medical record before the index hospitalization, consistent with the KDIGO Clinical Practice Guideline for the Evaluation and Management of CKD [11]. CKD was not newly assigned on the basis of serum creatinine measurements obtained during the index hospitalization. Patients receiving maintenance dialysis for end-stage kidney disease were excluded.

Microbiological data, including sputum and/or blood culture results obtained during hospitalization, and the requirement for temporary hemodialysis were also recorded retrospectively from the available medical records.

In addition, the length of hospital stay, need for intensive care, and in-hospital mortality were evaluated retrospectively from the available patient records.

### 2.5. Statistical Analysis

Statistical analyses were performed using IBM SPSS Statistics software (version 27; IBM Corp., Armonk, NY, USA). The normality of distribution for continuous variables was assessed using the Kolmogorov–Smirnov test. Continuous variables were expressed as mean ± standard deviation or median (interquartile range), depending on their distribution, whereas categorical variables were presented as number and percentage. For comparisons between two groups, Student’s *t*-test was used for normally distributed continuous variables, and the Mann–Whitney U test was used for variables not showing normal distribution. The chi-square test and, when appropriate, Fisher’s exact test were used for comparisons of categorical variables. For pairwise comparisons among KDIGO stages, the Mann–Whitney U test was used for continuous variables and Fisher’s exact test for categorical variables, as appropriate. *p*-values from pairwise comparisons were adjusted using the Bonferroni correction for multiple testing. Univariable logistic regression analyses were initially performed to evaluate associations between candidate clinical and laboratory variables and AKI. A multivariable logistic regression model was subsequently constructed to evaluate factors independently associated with AKI. Age, diastolic blood pressure, respiratory rate, confusion, DM, CKD, BUN, serum albumin, and RDW were entered simultaneously into the model using the Enter method. No automated stepwise variable-selection procedure was applied. Age was retained in the model as an a priori potential confounder irrespective of statistical significance. The CURB-65 score was not included in the multivariable model because its components overlap with several variables entered individually into the model, including age, respiratory rate, blood pressure, and BUN. Because CKD was uncommon in the non-AKI group and conventional maximum-likelihood estimates could therefore be affected by sparse-data bias, Firth penalized logistic regression was additionally performed as a sensitivity analysis using the same nine covariates included in the primary multivariable model. Profile penalized-likelihood 95% confidence intervals and penalized likelihood-ratio *p*-values were reported for the Firth model.

In an additional exploratory analysis, the admission BUN-to-creatinine ratio was calculated using simultaneously obtained admission BUN and serum creatinine values and was evaluated both univariably and in a multivariable model in which the BUN-to-creatinine ratio replaced BUN while the remaining covariates were unchanged.

Analyses were performed using available cases, and missing values were not imputed. Odds ratios (ORs) with 95% confidence intervals (CIs) were reported. All tests were two-sided, and a *p*-value of <0.05 was considered statistically significant.

## 3. Results

A total of 161 patients were included in the study, with a mean age of 70.9 ± 15.1 years. Of them, 110 (68.3%) were male, and 51 (31.7%) were female. For radiological confirmation of CAP, thoracic computed tomography was used in most patients (*n* = 143, 88.8%).

AKI was identified in 60 patients (37.3%). Of these, 43 patients (71.7%) fulfilled the KDIGO criteria for AKI at hospital admission, whereas 17 patients (28.3%) developed AKI during hospitalization after admission to the general ward. The median interval between the baseline serum creatinine measurement and hospital admission was 103 days (IQR, 28–316 days; range, 9–358 days). Among the 60 patients with AKI, 54 (90.0%) had a ≥1.5-fold increase in serum creatinine relative to baseline at one or more assessed time points, and 24 (40.0%) had a ≥0.3 mg/dL increase between admission and the subsequent 48 h; these criteria were not mutually exclusive. At the individual-patient level, the median peak-to-baseline serum creatinine ratio was 1.67 (IQR, 1.53–2.14), whereas the median admission-to-baseline serum creatinine ratio was 1.53 (IQR, 1.39–1.72). Hemodialysis was performed at least once in 8 of the 60 patients with AKI (13.3%). None of these patients remained dialysis-dependent at hospital discharge. During hospitalization, 43 patients (26.7%) initially admitted to the general ward subsequently required transfer to the intensive care unit. Overall, 31 patients (19.3%) died during hospitalization. The comparison of demographic characteristics, clinical findings, and laboratory parameters of patients with and without AKI is presented in Table 1.

To identify the microbiological etiology of CAP, sputum and/or blood cultures were obtained from 157 of the 161 patients. Positive microbiological growth was detected in 21 patients (13.4%). Culture results were available for 58 patients with AKI and 99 patients without AKI. Microorganisms were isolated in 5 of the 58 patients with AKI (8.6%) and in 16 of the 99 patients without AKI (16.2%), with no significant difference between the groups (*p* = 0.180).

In a post hoc exploratory analysis, the association of hypotension and acidosis at hospital admission with AKI was evaluated. Using the prespecified definition of systolic blood pressure < 90 mmHg and/or diastolic blood pressure ≤ 60 mmHg, AKI was identified in 20 of 44 patients (45.5%) with hypotension and in 40 of 117 patients (34.2%) without hypotension; this difference was not statistically significant (*p* = 0.188). Similarly, AKI was present in or developed in 14 of 25 patients (56.0%) with acidosis and in 46 of 136 patients (33.8%) without acidosis (*p* = 0.035).

The mean age of patients with AKI was significantly higher than that of patients without AKI (75.6 ± 12.5 vs. 68.0 ± 15.8 years; *p* = 0.002). The length of hospital stay was significantly longer in the AKI group (10.0 [IQR, 7.0–16.5] vs. 7.0 [IQR, 6.0–12.0] days; *p* = 0.038). In addition, 22 of 60 patients with AKI (36.7%) and 21 of 101 patients without AKI (20.8%) required intensive care (*p* = 0.028). In-hospital mortality was numerically higher in patients with AKI than in those without AKI (23.3% vs. 16.8%); however, the difference was not statistically significant (*p* = 0.312).

Admission diastolic blood pressure was significantly lower in patients with AKI (68 ± 11 vs. 72 ± 10 mmHg; *p* = 0.017), whereas admission mean arterial pressure did not differ significantly between the groups (*p* = 0.281). Respiratory rate was higher (22.0 ± 4.0 vs. 20.5 ± 3.2 breaths/min; *p* = 0.009), and confusion was more frequent (25.0% vs. 4.0%; *p* < 0.001) in patients with AKI. CURB-65 scores, recalculated using the standard criteria, were also higher in the AKI group (2.43 ± 1.13 vs. 1.43 ± 0.96; *p* < 0.001). No significant difference was found between the groups in contrast media exposure or ACE/ARB and diuretic use. Among antibiotic exposures recorded during hospitalization, penicillin exposure (48.3% vs. 29.7%; *p* = 0.018) and vancomycin exposure (15.0% vs. 4.0%; *p* = 0.017) were more frequent in patients with AKI, whereas cephalosporin, quinolone, and macrolide exposure did not differ significantly between groups. Because these exposures were recorded during hospitalization, these findings are descriptive and do not establish a temporal relationship with AKI. DM was more frequent in patients with AKI than in those without AKI (38.3% vs. 19.8%; *p* = 0.010), as was pre-existing CKD (31.7% vs. 3.0%; *p* < 0.001).

Evaluation of laboratory parameters revealed significantly higher BUN (33.6 [25.2–44.6] vs. 20.1 [15.9–26.2] mg/dL; *p* < 0.001) and RDW (15.8 [14.3–17.9]% vs. 14.9 [13.8–16.4]%; *p* = 0.015) values in patients with AKI. Serum albumin did not differ significantly between groups (31.4 ± 6.1 vs. 32.8 ± 5.1 g/L; *p* = 0.117). Potassium was higher (*p* = 0.013) and arterial pH was lower (*p* = 0.006) in the AKI group (Table 1).

According to the KDIGO classification, 38 of the 60 patients with AKI (63.3%) had stage I AKI, 10 (16.7%) had stage II AKI, and 12 (20.0%) had stage III AKI. Clinical outcomes according to KDIGO stage are summarized in Table 2. After Bonferroni adjustment for multiple pairwise comparisons, patients with stage III AKI had higher rates of ICU requirement (66.7% vs. 23.7%; adjusted *p* = 0.037) and in-hospital mortality (50.0% vs. 13.2%; adjusted *p* = 0.042) than patients with stage I AKI. Hemodialysis was required in 8 of 12 patients (66.7%) with stage III AKI and in none of the patients with stage I or stage II AKI; the differences were significant for stage III versus stage I (adjusted *p* < 0.001) and stage III versus stage II (adjusted *p* = 0.005). No pairwise difference in length of hospital stay remained statistically significant after Bonferroni adjustment.

Univariable logistic regression analysis was performed to evaluate factors associated with AKI. Older age, lower diastolic blood pressure, higher respiratory rate, confusion, DM, CKD, higher BUN, RDW, and potassium levels, and lower pH were significantly associated with AKI (Table 3). Serum albumin and total protein were not significantly associated with AKI. The recalculated CURB-65 score was also associated with AKI (OR 2.598 per 1-point increase, 95% CI 1.759–3.835; *p* < 0.001). For pH, the OR was expressed per 0.1-unit increase (OR 0.520, 95% CI 0.321–0.843; *p* = 0.008).

For the multivariable logistic regression analysis, the prespecified variables age, diastolic blood pressure, respiratory rate, confusion, DM, CKD, BUN, serum albumin, and RDW were entered simultaneously using the Enter method, with no automated stepwise variable elimination. Age was retained as an a priori potential confounder, and the CURB-65 score was not included because of overlap with variables already entered individually into the model [9]. In the conventional multivariable logistic regression model, confusion (adjusted OR 5.032, 95% CI 1.012–25.023; *p* = 0.048), pre-existing CKD (adjusted OR 6.003, 95% CI 1.281–28.132; *p* = 0.023), and higher BUN levels (adjusted OR 1.095 per 1 mg/dL increase, 95% CI 1.046–1.146; *p* < 0.001) remained independently associated with AKI (Table 4). DM was no longer independently associated with AKI after adjustment (adjusted OR 1.874, 95% CI 0.732–4.800; *p* = 0.190).

Because of the sparse distribution of CKD, Firth penalized logistic regression was additionally performed as a sensitivity analysis using the same covariates. The principal findings were retained: confusion (Firth OR 4.273, 95% profile-likelihood CI 1.026–21.506; *p* = 0.046), CKD (Firth OR 4.751, 95% profile-likelihood CI 1.252–22.946; *p* = 0.021), and BUN (Firth OR 1.085 per 1 mg/dL increase, 95% profile-likelihood CI 1.042–1.138; *p* < 0.001) remained independently associated with AKI (Table 5). The effect estimate for CKD was attenuated in the penalized model compared with the conventional logistic regression model, while the overall pattern of independently associated factors remained unchanged. DM remained non-significant in the Firth model (OR 1.792, 95% profile-likelihood CI 0.723–4.393; *p* = 0.204).

In an exploratory analysis, the admission BUN-to-creatinine ratio did not differ significantly between patients with and without AKI (median 21.52 [IQR, 16.93–27.70] vs. 21.10 [IQR, 16.69–29.30]; *p* = 0.548). The ratio was not associated with AKI in univariable analysis (OR 0.988, 95% CI 0.961–1.015; *p* = 0.372) and remained non-significant when substituted for BUN in the multivariable model (adjusted OR 0.976, 95% CI 0.940–1.013; *p* = 0.194).

## 4. Discussion

This study evaluated the frequency of AKI and the associated clinical factors in patients hospitalized due to CAP and revealed five key findings:

(a) AKI was identified in 37.3% of patients hospitalized with CAP.
(b) Length of hospital stay and need for intensive care were significantly higher in patients with AKI.
(c) Patients with AKI had significantly higher BUN and RDW levels, whereas serum albumin levels did not differ significantly between the groups.
(d) The presence of DM and CKD was significantly more common in patients with AKI.
(e) In multivariable logistic regression analysis, confusion, pre-existing CKD, and elevated BUN levels were independently associated with AKI, whereas DM was not independently associated after adjustment. These associations were retained in the Firth penalized logistic regression sensitivity analysis.

The AKI frequency of 37.3% observed in the present study indicates that AKI is a common clinical complication among hospitalized patients with CAP. The relatively high frequency of AKI in the present study may partly reflect the advanced age profile of the patient population and the high burden of accompanying comorbid diseases. Reduced renal reserve in older age and the systemic inflammatory response triggered by infection may increase susceptibility to renal dysfunction. Chen et al. reported AKI in 827 (28.7%) of 2883 patients with CAP with a median age of 76 years [12]. The researchers developed a prognostic nomogram incorporating albumin, acute respiratory failure, CURB-65 score, cystatin C, and white blood cell count [12]. The frequency of AKI observed in our cohort was somewhat higher than that reported by Chen et al., which may be related to differences in patient characteristics, comorbidity burden, AKI ascertainment, and study populations. Previous studies have evaluated AKI across heterogeneous CAP populations, including both general hospitalized cohorts and critically ill patients. Our study specifically included patients initially admitted to the general ward and evaluated routinely available clinical and laboratory variables. In the present cohort, serum albumin levels did not differ significantly between patients with and without AKI and were not associated with AKI in either univariable or multivariable analysis. Akram et al. reported an AKI on admission in approximately 18% of patients hospitalized with CAP and showed that greater pneumonia severity, higher CRP levels, and prior ACE inhibitor or angiotensin II receptor blocker use were independently associated with AKI [7]. Importantly, their study specifically evaluated AKI already present at admission, which is relevant to our cohort, in which most AKI cases also fulfilled the diagnostic criteria at hospital presentation. In a multicenter prospective cohort study conducted by Murugan et al., AKI was reported in 631 of 1836 hospitalized patients with CAP (34.4%), a frequency broadly similar to that observed in our cohort [3].

In a prospective observational study, Latief et al. found that AKI developed in 27.6% of 210 patients with CAP [13]. They showed that comorbidities such as CKD and coronary artery disease were more common in patients who developed AKI and that serum albumin levels were lower and BUN levels were higher in these patients [13]. These findings are partly consistent with our results, particularly regarding CKD and elevated BUN; however, we did not observe a significant difference in serum albumin. Thus, although hypoalbuminemia has been reported in other pneumonia-associated AKI cohorts, this finding was not reproduced in our study.

In a recent large-cohort study by Ma et al., AKI was reported in 15.8% of 6371 patients with CAP [14]. The researchers showed that diastolic blood pressure and serum albumin levels were lower and BUN levels were higher in patients with AKI [14]. Although the AKI frequency observed in the present study was higher, our findings were concordant with respect to lower diastolic blood pressure and higher BUN levels, whereas the significant albumin difference observed by Ma et al. was not reproduced in our cohort. Although admission diastolic blood pressure was statistically lower in patients with AKI, admission mean arterial pressure did not differ significantly between the AKI and non-AKI groups. Furthermore, the exploratory analysis based on the prespecified hypotension definition did not demonstrate a significant difference in AKI frequency according to the presence of hypotension. Therefore, the isolated difference in admission diastolic blood pressure should not be interpreted as evidence of clinically meaningful hypotension or impaired renal perfusion. BUN is not a kidney-specific marker and should therefore be interpreted cautiously in the context of acute systemic illness. Therefore, BUN should be interpreted as a nonspecific biomarker reflecting both renal function and overall disease severity rather than a kidney-specific marker. Moreover, because BUN was measured concurrently with the assessment of AKI, its association in the present study should not be interpreted as evidence of temporal prediction of subsequent AKI development. Consistent with this cautious interpretation, the admission BUN-to-creatinine ratio was not associated with AKI in either univariable or multivariable exploratory analyses, suggesting that the observed association with BUN was not reproduced when BUN was expressed relative to admission creatinine.

More recently, Shi et al. prospectively evaluated 162 patients with severe CAP at high altitude and reported AKI in 74 patients (45.7%) [15]. Multivariable analysis demonstrated that septic shock, impaired oxygenation, BUN ≥ 7 mmol/L, albumin < 30 g/L, and reduced left ventricular ejection fraction were independently associated with AKI [15]. Although their high-altitude severe-CAP population differed substantially from our general-ward cohort, the independent association between elevated BUN and AKI was concordant with our findings. Direct comparison of AKI frequencies between the two studies should therefore be made cautiously.

DM was more frequent among patients with AKI and was associated with AKI in univariable analysis; however, this association was no longer significant after multivariable adjustment. Accordingly, DM should be regarded as a marker of comorbidity burden in the present cohort rather than an independently associated factor.

In contrast, pre-existing CKD remained independently associated with AKI in both the conventional multivariable model and the Firth penalized logistic regression sensitivity analysis. However, the association between CKD and AKI should be interpreted with caution, as the relatively small number of patients with CKD in the non-AKI group resulted in a wide confidence interval and therefore limited the precision of the estimated effect size. The attenuation of the CKD effect estimate from an adjusted OR of 6.003 in the conventional model to a Firth OR of 4.751 further supports cautious interpretation of the magnitude of this association, although statistical significance was retained.

Because most AKI cases in our cohort (43/60, 71.7%) fulfilled the KDIGO criteria at hospital admission, the observed associations should be interpreted as factors associated with the presence of AKI rather than predictors of incident AKI during hospitalization. This temporal distribution also limits interpretation of post-admission hemodynamic changes and medication exposures as potential determinants of AKI in the overall cohort. In the final analysis, baseline renal function was defined uniformly using the closest clinically stable pre-admission serum creatinine value obtained within 7–365 days before hospitalization; therefore, all AKI classifications and subsequent analyses were based on this single prespecified baseline definition.

In recent years, studies evaluating AKI in patients with CAP, particularly those admitted to intensive care units, have attracted increasing attention. Almutairi et al. reported an AKI incidence of 46% in 1536 mechanically ventilated patients with CAP followed in the intensive care unit [16]. Crude in-hospital mortality increased across AKI stages; however, AKI stage was not independently associated with in-hospital mortality after multivariable adjustment. Older age, vasopressor use, lower Glasgow Coma Scale score, lower PaO_2_/FiO_2_ ratio and platelet count, and higher bilirubin, lactate, and INR were associated with mortality [16].

Ji et al. studied 1795 ICU patients who already had both CAP and AKI and developed machine-learning models for 28-day mortality [17]. BUN was among the important features associated with mortality prediction in that population; therefore, this study provides information on prognosis after AKI rather than on factors associated with AKI occurrence [17].

Zhang and Song evaluated 267 mechanically ventilated patients with severe pneumonia, of whom 77 (28.8%) developed AKI [18]. Albumin levels were lower and BUN levels were higher in patients with AKI; however, after multivariable adjustment, lower albumin remained independently associated with AKI whereas BUN did not [18]. Unlike these studies, which predominantly included critically ill patients requiring intensive-care-level support, our study included patients initially admitted to the general ward. This difference in study population should be considered when comparing AKI incidence and associated clinical factors across studies. Taken together, these studies illustrate that the associations of BUN and albumin with AKI may differ according to population severity, clinical setting, and statistical adjustment [15,16,17,18].

However, in our cohort, CRP levels did not differ significantly between patients with and without AKI, and admission MAP was also similar between the groups. Therefore, the present data do not support greater systemic inflammatory activity, as reflected by CRP, or clinically meaningful hemodynamic impairment as distinguishing features of AKI in this cohort. Although RDW was significantly associated with AKI in the univariable analysis, this association was not maintained after multivariable adjustment. Therefore, in the present cohort, RDW may reflect overall illness-related biological disturbance rather than an independent factor associated with AKI.

Beyond CAP, studies have also reported associations between AKI and adverse outcomes in other inflammatory and infectious diseases. Aristizabal et al., in a study of 1722 patients diagnosed with COVID-19, reported AKI in 30.7% of patients and substantially higher in-hospital mortality among patients with AKI than among those without AKI (58.1% vs. 19.6%) [19].

Morcos et al. reported AKI in 13.6% of patients hospitalized with chronic obstructive pulmonary disease (COPD) exacerbation and found that AKI was associated with higher in-hospital mortality after multivariable adjustment (OR 2.417) [20]. Truong et al. showed that among patients hospitalized for COPD exacerbation, the incidence of AKI was higher when bacterial pneumonia was present (16.8% vs. 10.3%) and that AKI was independently associated with higher in-hospital mortality (OR 2.33) [21]. The common finding across these studies is that AKI is associated with poor clinical outcomes and increased mortality. In the present study, in-hospital mortality was numerically higher in patients with AKI than in those without AKI (23.3% vs. 16.8%), but the difference was not statistically significant. Therefore, our data do not establish an overall mortality association between AKI and non-AKI groups. Nevertheless, within the AKI subgroup, patients with stage III AKI had higher in-hospital mortality than those with stage I AKI after adjustment for pairwise comparisons, suggesting that greater AKI severity was associated with worse short-term outcome within this cohort.

The clinical relevance of AKI accompanying pneumonia may also extend beyond the index hospitalization. Chawla et al. reported that patients hospitalized with both pneumonia and AKI had worse post-discharge major adverse kidney outcomes, including death, chronic dialysis, and persistent loss of renal function, than patients with either condition alone [22].

AKI in patients hospitalized with CAP is considered to result from multifactorial mechanisms. Inflammatory mediator activation and renal microcirculatory dysfunction have been implicated in the pathogenesis of AKI in patients with acute lung injury and critical illness [23,24]. However, CRP concentrations were similar in the AKI and non-AKI groups in the present cohort (127 vs. 140 mg/L; *p* = 0.569). Accordingly, our findings do not demonstrate greater systemic inflammatory activity in patients with AKI, and the inflammatory mechanisms described above should be regarded as biological mechanisms reported in the literature rather than mechanisms directly demonstrated by the present study. In addition, exposure to nephrotoxic drugs and preexisting comorbid diseases may contribute to AKI in the setting of acute infection. Although contrast exposure, ACE inhibitor/ARB use, diuretic therapy, and vancomycin exposure were evaluated in our study, contrast exposure and pre-admission ACE/ARB and diuretic use did not differ significantly between groups, whereas vancomycin exposure during hospitalization was more frequent in patients with AKI. Because antibiotic exposure was recorded during hospitalization and detailed information regarding dose, duration, and temporal relationship to AKI onset was not consistently available, the higher frequency of vancomycin exposure should not be interpreted as evidence that vancomycin caused AKI. Exposure to other potentially nephrotoxic medications, including aminoglycosides and nonsteroidal anti-inflammatory drugs, was also not consistently available because of the retrospective design. Similarly, detailed information regarding sepsis severity, septic shock, vasopressor therapy, mechanical ventilation, fluid balance, and the timing of antimicrobial therapy was not consistently available and therefore could not be incorporated into the present analyses.

### Limitations

The main limitations of the present study are its single-center and retrospective cohort design and the inherent potential for selection bias, residual confounding, and incomplete ascertainment associated with retrospective data collection. Although CAP diagnosis was based on documented clinical, laboratory, and radiological findings, some degree of diagnostic misclassification cannot be excluded. In addition, the exact onset of AKI before admission could not be determined in patients who already fulfilled KDIGO criteria at presentation. Urine output data were not reliably available; therefore, AKI ascertainment relied on serum creatinine criteria, with initiation of renal replacement therapy incorporated into stage 3 classification. Furthermore, because most AKI cases were already present at hospital admission, the observed associations should be interpreted as associations with AKI rather than predictors of incident AKI during hospitalization. Baseline renal function was retrospectively determined using the closest clinically stable pre-admission serum creatinine value obtained within 7–365 days and therefore remained dependent on the availability and timing of prior measurements. Serial hemodynamic data and detailed information on the timing, dose, and duration of antimicrobial and other potentially nephrotoxic exposures were not consistently available, limiting assessment of their temporal relationship with AKI. Although the multivariable model included nine prespecified covariates, the relatively limited number of AKI events may have reduced model precision, particularly for CKD. Firth penalized logistic regression was therefore performed as a sensitivity analysis and yielded consistent findings; nevertheless, the wide confidence intervals for confusion and CKD indicate limited precision. Other potentially relevant factors, including sepsis severity, vasopressor use, mechanical ventilation, fluid balance, and additional nephrotoxic exposures, could not be consistently evaluated. Information regarding renal recovery, persistent kidney dysfunction, and post-discharge renal outcomes was also unavailable. Finally, the single-center design and restriction to patients initially admitted to the general ward may limit generalizability. Therefore, the findings require validation in larger prospective multicenter studies.

## 5. Conclusions

In conclusion, AKI was common among patients hospitalized with CAP. Patients with AKI had significantly longer hospital stays and a greater need for intensive care. Confusion, pre-existing CKD, and elevated BUN levels were independently associated with AKI in the multivariable analysis, and these associations were retained in the Firth penalized logistic regression sensitivity analysis. DM was associated with AKI in univariable analysis but was not independently associated after multivariable adjustment. These findings should be interpreted as associations with AKI rather than predictors of subsequent AKI development, particularly because most AKI cases were already present at hospital admission. Accordingly, the present results may help characterize the clinical profile of hospitalized patients with CAP and concomitant AKI but should not be interpreted as establishing causality or predicting incident AKI. Future prospective multicenter studies are warranted to validate these findings and to clarify the temporal relationship between clinical and laboratory factors and incident AKI in patients hospitalized with CAP.

## Figures and Tables

**Table 1:** Demographic, clinical, and laboratory characteristics of patients with and without AKI.

	Patients with AKI (*n*: 60)	Patients Without AKI (*n*: 101)	*p*-Value
Age (years)	75.6 ± 12.5	68.0 ± 15.8	0.002
Age ≥ 65 *n* (%)	50 (83.3%)	61 (60.4%)	0.002
Sex (Female/Male)	16/44	35/66	0.292
Length of hospital stay (days)	10.0 (7.0–16.5)	7.0 (6.0–12.0)	0.038
Smoking *n* (%)	18 (31.6%)	28 (29.8%)	0.817
Intensive care hospitalization *n* (%)	22 (36.7%)	21 (20.8%)	0.028
Mortality *n* (%)	14 (23.3%)	17 (16.8%)	0.312
CURB-65 score	2.43 ± 1.13	1.43 ± 0.96	<0.001
CURB-65 score			
0 point (*n*: 20)	0 (0.0%)	20 (19.8%)	<0.001
1 point (*n*: 39)	10 (16.7%)	29 (28.7%)	
2 point (*n*: 75)	31 (51.7%)	44 (43.6%)	
3 point (*n*: 10)	5 (8.3%)	5 (5.0%)	
4 point (*n*: 14)	11 (18.3%)	3 (3.0%)	
5 point (*n*: 3)	3 (5.0%)	0 (0.0%)	
Vital signs			
Confusion	15 (25.0%)	4 (4.0%)	<0.001
Admission SBP (mmHg)	121 ± 22	119 ± 20	0.589
Admission DBP (mmHg)	68 ± 11	72 ± 10	0.017
Admission MAP (mmHg)	85 ± 14	88 ± 12	0.281
Heart rate (beats/min)	96 ± 20	95 ± 15	0.682
Respiratory rate	22.0 ± 4.0	20.5 ± 3.2	0.009
Body temperature (°C)	36.61 ± 0.42	36.57 ± 0.41	0.572
Medication/Substance use *n* (%)			
Diuretics	18 (30%)	21 (20.8%)	0.187
ACE/ARB	11 (18.6%)	14 (13.9%)	0.421
Contrast agent	4 (6.7%)	7 (6.9%)	1.000
Penicillin	29 (48.3%)	30 (29.7%)	0.018
Cephalosporin	25 (41.7%)	58 (57.4%)	0.053
Quinolone	18 (30.0%)	20 (19.8%)	0.141
Macrolide	29 (48.3%)	55 (54.5%)	0.452
Vancomycin	9 (15.0%)	4 (4.0%)	0.017
Comorbidities *n* (%)			
HT	32 (53.3%)	50 (49.5%)	0.638
DM	23 (38.3%)	20 (19.8%)	0.010
CAD	23 (38.3%)	27 (26.7%)	0.124
CHF	18 (30.0%)	20 (19.8%)	0.141
COPD	16 (26.7%)	28 (28.0%)	0.855
Dementia	5 (8.3%)	8 (7.9%)	1.000
CVE	6 (10.2%)	8 (7.9%)	0.627
AF	15 (25%)	16 (15.8%)	0.154
CKD	19 (31.7%)	3 (3.0%)	<0.001
Hemogram			
Hb (g/dL)	11.4 ± 2.3	11.6 ± 2.1	0.459
Leukocyte count (cells/mm^3^)	10,765 (7658–16,132)	10,325 (7315–12,992)	0.286
Neutrophil count (cells/mm^3^)	9450 (6070–13,432)	8100 (5698–11,242)	0.110
Lymphocyte count (cells/mm^3^)	985 (670–1460)	1170 (688–1660)	0.252
NLR	10.6 (4.9–16.7)	7.3 (4.3–12.7)	0.065
RDW (%)	15.8 (14.3–17.9)	14.9 (13.8–16.4)	0.015
Platelets count (×10^3^/mm^3^)	253 (178–318)	256 (194–339)	0.481
Biochemical tests			
Baseline serum creatinine (mg/dL)	1.02 (0.87–1.16)	0.79 (0.65–0.90)	<0.001
Admission serum creatinine (mg/dL)	1.56 (1.21–1.91)	0.91 (0.69–1.03)	<0.001
48-h serum creatinine (mg/dL)	1.48 (1.14–1.93)	0.82 (0.66–1.00)	<0.001
Day-7 serum creatinine (mg/dL)	1.33 (1.10–1.76)	0.80 (0.68–0.98)	<0.001
Peak serum creatinine (mg/dL)	1.75 (1.52–2.19)	0.94 (0.78–1.14)	<0.001
BUN (mg/dL)	33.6 (25.2–44.6)	20.1 (15.9–26.2)	<0.001
CRP (mg/L)	127 (85–212)	140 (90–215)	0.569
T.Protein (g/L)	63.3 ± 9.3	64.7 ± 7.7	0.304
Albumin (g/L)	31.4 ± 6.1	32.8 ± 5.1	0.117
ALT (U/L)	17 (13–30)	19 (12–34)	0.597
AST (U/L)	22 (16–38)	24 (18–32)	0.709
LDH (U/L)	244 (201–300)	220 (180–302)	0.269
Na (mmol/L)	137 (135–140)	137 (134–139)	0.841
K (mmol/L)	4.25 ± 0.64	4.00 ± 0.58	0.013
Arterial blood gases			
pH	7.39 ± 0.08	7.42 ± 0.06	0.006
pCO2	41 ± 10	42 ± 9	0.787
pO2	60 ± 14	62 ± 16	0.511
sO2	87 ± 9	88 ± 10	0.625
Lactate	1.5 (1.1–2.2)	1.4 (1.1–2.0)	0.636

Abbreviations: AKI, acute kidney injury; ACE, angiotensin-converting enzyme; ARB, angiotensin II receptor blocker; AF, atrial fibrillation; ALT, alanine aminotransferase; AST, aspartate aminotransferase; BUN, blood urea nitrogen; CAD, coronary artery disease; CHF, congestive heart failure; CKD, chronic kidney disease; COPD, chronic obstructive pulmonary disease; CRP, C-reactive protein; CURB-65, Confusion, Urea, Respiratory rate, Blood pressure, Age ≥ 65 years; CVE, cerebrovascular event; DBP, diastolic blood pressure; DM, diabetes mellitus; Hb, hemoglobin; HT, hypertension; LDH, lactate dehydrogenase; MAP, mean arterial pressure; Na, sodium; NLR, neutrophil-to-lymphocyte ratio; RDW, red cell distribution width; SBP, systolic blood pressure. Data presentation and analysis: Data are presented as *n* (%), mean ± SD, or median (IQR), as appropriate. Continuous variables were compared using Student’s *t*-test or the Mann–Whitney U test; categorical variables were compared using Pearson’s chi-square or Fisher’s exact test, as appropriate. Analyses used available cases; missing values were not imputed. Renal and CURB-65 definitions: Baseline serum creatinine was the closest clinically stable pre-admission value obtained within 7–365 days before hospitalization. Peak serum creatinine was recalculated as the highest of admission, 48-h, and day-7 values. CURB-65 was recalculated patient-by-patient using standard criteria, with BUN > 19.6 mg/dL corresponding to urea > 7 mmol/L.

**Table 2:** Comparison of clinical outcomes according to KDIGO stage.

Variable	Stage I (*n* = 38 63.3%)	Stage II (*n* = 10 16.7%)	Stage III (*n* = 12 20.0%)	*p*-Value
Length of hospital stay (days)	9.5 (6.3–15.5)	7.0 (7.0–9.0)	16.0 (10.0–27.8)	1.000 * 0.132 ** 0.110 ***
Intensive care requirement, *n* (%)	9 (23.7%)	5 (50.0%)	8 (66.7%)	0.390 * 0.037 ** 1.000 ***
Hemodialysis requirement, *n* (%)	0 (0%)	0 (0%)	8 (66.7%)	— * <0.001 ** 0.005 ***
In-hospital mortality, *n* (%)	5 (13.2%)	3 (30.0%)	6 (50.0%)	1.000 * 0.042 ** 1.000 ***

Abbreviations: KDIGO, Kidney Disease: Improving Global Outcomes. * Stage I vs. Stage II; ** Stage I vs. Stage III; *** Stage II vs. Stage III. Pairwise comparisons for length of hospital stay were performed using the Mann–Whitney U test, whereas categorical outcomes were compared using two-sided Fisher’s exact test. *p*-values for all pairwise comparisons were adjusted using the Bonferroni correction. The Stage I vs. Stage II comparison for hemodialysis requirement was not estimable because no events occurred in either group.

**Table 3:** Univariable logistic regression analysis of factors associated with AKI.

Variables	OR	95% Confidence Interval	*p*-Value
Age (years)	1.040	1.013–1.066	0.003
Age (≥65)	3.279	1.492–7.206	0.003
Diastolic blood pressure	0.964	0.934–0.994	0.020
Respiratory rate	1.129	1.028–1.239	0.011
Confusion	8.083	2.539–25.740	<0.001
CURB-65	2.598	1.759–3.835	<0.001
DM	2.518	1.232–5.143	0.011
CKD	15.138	4.247–53.963	<0.001
BUN	1.122	1.078–1.167	<0.001
T.Protein	0.980	0.943–1.019	0.303
Albumin	0.954	0.900–1.012	0.119
RDW	1.168	1.039–1.313	0.009
K	1.976	1.142–3.421	0.015
pH (per 0.1-unit increase)	0.520	0.321–0.843	0.008

Abbreviations: AKI, acute kidney injury; BUN, blood urea nitrogen; CKD, chronic kidney disease; CURB-65, Confusion, Urea, Respiratory rate, Blood pressure, Age ≥ 65 years; DM, diabetes mellitus; OR, odds ratio; RDW, red cell distribution width; K, potassium. For pH, the OR represents the change in odds associated with a 0.1-unit increase. For other continuous variables, ORs represent a 1-unit increase unless otherwise specified. All analyses included the complete cohort (*n* = 161).

**Table 4:** Multivariable logistic regression analysis of factors independently associated with AKI.

Variables	Adjusted OR	95% Confidence Interval	*p*-Value
Age (years)	1.009	0.977–1.041	0.598
Diastolic blood pressure	0.998	0.953–1.044	0.929
Respiratory rate	1.100	0.966–1.252	0.150
Confusion	5.032	1.012–25.024	0.048
DM	1.874	0.732–4.800	0.190
CKD	6.003	1.281–28.133	0.023
BUN	1.095	1.046–1.146	<0.001
Albumin	0.972	0.897–1.053	0.486
RDW	1.096	0.937–1.283	0.251

Abbreviations: AKI, acute kidney injury; BUN, blood urea nitrogen; CI, confidence interval; CKD, chronic kidney disease; DM, diabetes mellitus; OR, odds ratio; RDW, red cell distribution width. ORs for continuous variables represent a 1-unit increase. All covariates were entered simultaneously using the Enter method. No automated stepwise variable-selection procedure was applied.

**Table 5:** Firth penalized logistic regression sensitivity analysis of factors independently associated with AKI.

Variable	Firth OR	95% Profile-Likelihood CI	*p*-Value
Age (years)	1.007	0.978–1.039	0.635
Diastolic blood pressure	0.999	0.956–1.043	0.957
Respiratory rate	1.089	0.963–1.235	0.175
Confusion	4.273	1.026–21.506	0.046
DM	1.792	0.723–4.393	0.204
CKD	4.751	1.252–22.946	0.021
BUN	1.085	1.042–1.138	<0.001
Albumin	0.974	0.902–1.051	0.492
RDW	1.092	0.938–1.267	0.254

Abbreviations: AKI, acute kidney injury; BUN, blood urea nitrogen; CI, confidence interval; CKD, chronic kidney disease; DM, diabetes mellitus; OR, odds ratio; RDW, red cell distribution width. Firth penalized logistic regression was performed as a sensitivity analysis using the same covariates as the primary multivariable model. Confidence intervals are profile penalized-likelihood intervals, and *p*-values are based on penalized likelihood-ratio tests.

## Data Availability

All data generated or analyzed during this study are included in this article. The data will be available upon reasonable request (contact: omer.topaloglu@erdogan.edu.tr).
